# Increased nodular P level induced by intercropping stimulated nodulation in soybean under phosphorus deficiency

**DOI:** 10.1038/s41598-022-05668-z

**Published:** 2022-02-07

**Authors:** Xiaomin Qin, Haonan Pan, Jingxiu Xiao, Li Tang, Yi Zheng

**Affiliations:** 1 Present Address: College of Resources and Environmental Science, Yunnan Agricultural University, Kunming, 650201 China; 2 Guangxi South Subtropical Agricultural Science Research Institute, Chongzuo, 532200 China; 3 Yunnan Open University, Kunming, 650599 China; 4 Present Address: College of Resources and Environmental Science, Yunnan Agricultural University, Kunming, 650201 China

**Keywords:** Rhizobial symbiosis, Plant physiology

## Abstract

Low P availability is a vital constraint for nodulation and efficient N_2_ fixation of legume, including soybean. To elucidate the mechanisms involved in nodule adaption to low P availability under legume/cereal intercropping systems, two experiments consisting of three cropping patterns (monocropped soybean, monocropped maize, soybean/maize intercropping) were studied under both sufficient- and deficient-P levels. Our results demonstrated that intercropped soybean with maize showed a higher nodulation and N_2_ fixation efficiency under low P availability than monocropped soybean as evidenced by improvement in the number, dry weight and nitrogenase activity of nodules. These differences might be attributed to increase in P level in intercropping-induced nodules under low P supply, which was caused by the elevated activities of phytase and acid phosphatases in intercropping-induced nodules. Additionally, the enhanced expression of phytase gene in nodules supplied with deficient P level coincided with an increase in phytase and acid phosphatase activities. Our results revealed a mechanism for how intercropped maize stimulated nodulation and N_2_ fixation of soybean under P deficient environments, where enhanced synthesis of phytase and acid phosphatases in intercropping-induced nodules, and stimulated nodulation and N_2_ fixation.

## Introduction

Phosphorus is one of the most important macronutrients for plant growth and development, and plays a vital role in increasing crop yield^1,2^. Unfortunately, low P availability in many agricultural soils is nearly universal, because P massively precipitates to form various unavailable complexes with metals, such as iron and aluminium in acidic soil^3,4^. Furthermore, P deficiency in soils in agriculture production relies on continual supply of P fertilizers which will trigger greater environmental risks and P resource loss. Additionally, mineral P resources are non-renewable and global high-grade rock phosphates are estimated to be depleted within 100 years^5,6^. Consequently, the influence of limiting the input of P to agricultural ecosystem is going to become a global problem.

Legume, as a key part of sustainable agro-ecosystem, plays an important role in effective management of fertilizers and improving soil health^7^. However, low P availability in soils is one of the most obvious abiotic factors that limits the growth and productivity of legume, because of the decrease in N_2_ fixation^8,9^. For nodules in particular, the role of P is crucial in the metabolic reactions that drive symbiotic N_2_ fixation^10–12^. A reduction of P levels in nodules under P starvation, resulting in significant declines in nodule formation, nitrogenase activity and N_2_ fixation^13,14^. Thus, the symbiotic N_2_ fixation efficiency of nodulated legume is most likely determined by P level within the nodules^15^.

Intercropping, growing simultaneously two or more crop species within the same space during a considerable part of their lifecycle^16^; and legume/cereal intercropping is a classical case. A large number of results derived from field and pot experiments have proved that intercropping of legume and cereal could enhance the efficient utilization of phosphorus and yield through interspecific facilitation, even under phosphorus deficient conditions^17–20^. Additionally, evidences had accumulated recently that the practice of intercropped legume with cereal promoted the nodulation and N_2_ fixation of legume by interspecific facilitation, and resulted in increased N uptake of associated cereal^20–23^. Considering the interspecific facilitation, we hypothesized that P level in nodules of legume under P stressful environment would be stimulated by intercropping.

Acid phosphatase (APase), as a unique group of enzymes, plays an important role in internal P homeostasis by the production, transport and remobilization of phosphorus^24^. Particularly phytase, as one of the most interesting classes of APase, catalyzes the release of phosphate from phytate; and phytic acid is the principal storage form of phosphorus in tissues of higher plants^25,26^. Significant increase in synthesis and secretion of acid phosphatases in legume respond to P stress to stimulate effective utilization of internal and external phosphorus, particularly under legume/cereal intercropping patterns consisting of faba bean/maize and peanut/maize^27–29^. In legume nodules, phytase and acid phosphatase also had been observed, and it had been demonstrated that their elevated activities induced by P deficiency resulted in a higher nodular P level^30–32^. However, how intercropped cereal induced the synthesis of phytase and acid phosphatases in nodules by root–root interaction under P stressful environments and their specific role in affecting nodular P level remain poorly documented.

Soybean (*Glycine max* L.) widely is adopted to intercrop with other cereals in the world due to its superior ability in N_2_ fixation^33^. The intercropping of soybean and maize is widely practiced as a promising option in China, due to its outstanding advantages in improvement of nutrient (N and P) use efficiency and yield^34–36^. Much work on improvement of P content in nodules under P deficiency today were mainly focused on soybean grown alone^37–39^, but little information is available about the P level in nodules altered by intercropping of soybean and maize. In this context, in order to elucidate the interaction of P deficiency—nodular P level—nodulation under soybean/maize intercropping, we conducted experiments under both sufficient and deficient P levels to test the hypothesis that intercropped soybean and maize grown under P-deficient conditions could increase the P level in nodules. We also hypothesized that activities of acid phosphatase and phytase in nodules highly stimulated by intercropping of soybean and maize under P deficiency would result in elevated nodular P level.

## Materials and methods

### Materials and growth conditions

All experiments involving plants were carried out in accordance with relevant institutional, national, and international guidelines and legislation.

### Experimental design

#### Soil experiment

A pot experiment was conducted with three cropping patterns and two P supplies. Soybean or maize was grown alone as a monocropping pattern (monocropped soybean, monocropped maize), or intercropped with maize (soybean//maize) in soil supplied with 50 and 100 mg P kg^−1^ soil, corresponding to deficient-P and sufficient-P levels, respectively. There were six treatments with four replicates per treatment in this experiment.

The tested soil was collected from Xiaoshao experimental station in Kunming (102°99′E, 25°17′N), Yunnan province, China and it was a typical red soil with a strong capacity of phosphorus fixation. Soil properties were as follows: Olsen-P 4.77 mg kg^−1^, organic C 7.58 g kg^−1^, available N 30.87 mg kg^−1^, available K 125.44 mg kg^−1^ and pH 5.19 (the ratio of soil to distilled water was 1: 2.5). Each pot (24 cm height × 19.5 cm diameter) was filled with 10 kg of air-dried soil. To ensure that the nutrient supply was adequate for plant growth, 150 mg N kg^−1^ soil and 150 mg K kg^−1^ soil were also fertilized with basal nutrients. Urea, superphosphate, and potassium sulfate were applied in the soil experiment as N, P and K fertilizers.

The genotype of soybean (*Glycine max* L.) was Diandou-7(Authorized No.:2010017), and the genotype of maize (*Zea mays* L*.*) was Yunrui-88(Authorized No.: 2009012), provided by Yunnan Academy of Agricultural Sciences. The seeds of soybean and maize were surface sterilized with 75% (vol/vol) alcohol for 5 min and rinsed with sterilized distilled water and then, sterilized with 15% (vol/vol) H_2_O_2_ for 10 min and rinsed with sterilized distilled water, and then germinated in a growth chamber of 22 °C for 48 h under dark. Then, the germinated seeds of soybean or maize were uniformly planted in two rows in each pot in monocropped pattern, while intercropping designed with a row of soybean and a row of maize based on the replacement principle. All of the pots were arranged in a completely randomized block design and were re-randomized weekly during the experimental period. All soybean plants were inoculated with a suspension of ∼ 10^8^ cells mL^−1^ of rhizobium strains BNCC336406 (R. Bradyrhizobium japonicum (Kirchner) Jordan). The plants were watered every day to maintain field capacity (20–25%, w/w).

At 75 days (flowering stage), leaves, roots, and nodules were separately harvested for measuring dry weight, P content, nodule number and nodule dry weight. In addition, fractions of leaf, root, and nodule used for assays of enzyme activity, gene expression were frozen immediately in liquid N_2_, and stored at − 80 °C.

#### Hydroponic experiment

A hydroponic experiment was set up with three cropping patterns (soybean, maize and soybean/maize) and two P supplies (deficient-P and sufficient-P levels). There were six treatments arranged in a complete randomized block design with 4 replicates of each treatment.

The same genotypes of maize and soybean seeds were chosen and handled as in the soil experiment. After germination, the young seedlings of soybean were inoculated with a suspension of rhizobium strains BNCC336406 (∼10^8^ cells mL^−1^). The planting density of soybean and maize plants in monocropping or intercropping pattern was same as in the soil experiment.

Then, these seedlings of inoculated soybean and maize were transplanted into each 3 L-container filled with full nutrients containing 2000 μM Ca (NO_3_)_2_, 750 μM K_2_SO_4_, 650 μM MgSO_4_, 100 μM KCl, 0.1 μM H_3_BO_3_, 1 μM MnSO_4_, 0.1 μM CuSO_4_, 1 μM ZnSO_4_, 0.005 μM (NH4)_6_Mo_7_O_24_, 100 μM Fe-EDTA, and KH_2_PO_4_ was supplemented to the nutrient solution to a concentration of either 250 μM or 125 μM to establish sufficient or deficient P levels, respectively. An appropriate concentration of K_2_SO_4_ was added to the P-deficient solution to ensure an equal supply of K. The pH of nutrient solution was adjusted to 5.5 with HCl (0.01 mol·L^−1^). The nutrient solution was replaced two times every week until harvest. The oxygenation of nutrient solution was ensured by a permanent flow of 400 ml min^−1^ of compressed air. At 62 days (flowering stage), leaves, roots, and nodules were separately harvested and determined as in the soil experiment.

Both experiments were conducted in a glasshouse at Yunnan Agricultural University (YNAU), Kunming (latitude: 40°08′N, longitude: 102°48′E). In the soil experiment, the temperature in the glasshouse was maintained at 24–32 °C during the day and 15–18 °C at night, with 12–14 h daytime throughout the growth period. In hydroponic experiment, temperature was maintained at 25–30 °C during the day and 18–21 °C at night, with 14–16 h daytime.

## Measurements

### Plant biomass and P content

Shoots, roots and nodules were dried at 75 °C to a constant weight for dry weight determination. P content in shoots, roots and nodules were measured by the photometric method at 450 nm after digesting with a mixture of concentrated H_2_SO_4_ and H_2_O_2_.

### Nitrogenase activity

Nodule nitrogenase activity was measured by the acetylene reduction assay. Fresh nodules were placed in a closed gas reaction bottle and injected with 10 mL acetylene gas for 2 h at 28 °C. Thereafter, the ethylene was measured by gas chromatography (7820A GC system; Agilent Technologies). A standard curve for ethylene was developed according to the standard peak area of ethylene to calculate the ethylene content. Nitrogenase activity was calculated as milliliter ethylene h^−1^ g^−1^ nodule.

### Phytase and APase activities

APase and phytase were extracted from leaves, roots, and nodules by enzymic reagents according to the manufacturers’ recommendations (www.geruisi-bio.com, China). APase activity was defined as the amount of p-nitrophenol (PNP) produced by hydrolyzing 1 μmol of p-nitrophenyl phosphate (PNPP) per minute per gram of fresh sample at 37 °C. Phytase activity was calculated as 1 μmol of Pi released per minute per gram of fresh sample at 37 °C and pH5.5.

### RT-PCR of phytase gene in nodules

Total RNA was extracted from nodules and roots, using Trizol reagent according to the manual (SinoGene, China). First- strand cDNA was synthesized from 2 mg DNaseI treated RNA (Fermentas). The qRT-PCR analysis was conducted using 2 × SG Green monitored qRT-PCR (SinoGene, China) and a StepOnePLUS qRT-PCR system (USA). The gene expression analysis had three biological replications. Relative expression level was calculated from the ratio of expression levels of candidate genes to expression level of the housekeeping gene, TEFS1.

### Statistical analyses

Data in this study were reported as means ± standard deviation (SD) of the four replicates. Data were subjected to a one-way ANOVA, and significant differences between P levels and cropping patterns were measured by (LSD) test (P ≤ 0.05). All statistical analyses in this study were conducted with SPSS statistical software (SPSS version 19.0, IBM SPSS Inc., Chicago, IL, USA).

## Results

### Plants growth

Intercropping significantly increased biomass compared to monocropping at two levels of P supply in two experiments (Table 1). In soil and hydroponic experiments, total shoot and root dry matter (DM) accumulations in intercropped soybean supplied with deficient or sufficient P levels were significantly greater than that in monocropped soybean, respectively, and the shoot and root DM of intercropped maize were significantly enhanced compared to monocropped maize. A reduction in P supply resulted in a significant decrease in DM accumulation in shoots and roots in both maize and soybean. Root DM was more affected than shoot DM under deficient P conditions in soybean. This observation was particularly evident in soybean as evidenced by the significant decrease in the ratio of root to shoot when the P supply reduced.

**Table 1:** Growth of soybean and maize as affected by intercropping and P levels.

Experiments	Times (days)	Cropping patterns	Shoots biomass/g plant^−1^		Roots biomass/g plant^−1^		Root/shoot ratio	
			Deficient-P	Sufficient-P	Deficient-P	Sufficient-P	Deficient-P	Sufficient-P
Soil experiment	75d	MS	8.15^d^ ± 0.12	12.40^c^ ± 0.24	1.16^d^ ± 0.09	2.48^b^ ± 0.14	0.142^b^	0.200^a^
		IS	13.79^b^ ± 0.80	17.02^a^ ± 0.67	2.17^b^ ± 0.12	3.52^a^ ± 0.13	0.157^b^	0.207^a^
		MM	15.55^d^ ± 0.44	22.85^c^ ± 1.02	3.69^c^ ± 0.16	5.11^b^ ± 0.20	0.237^a^	0.224^a^
		IM	24.39^b^ ± 0.47	31.21^a^ ± 1.20	5.46^b^ ± 0.27	6.66^a^ ± 0.52	0.224^a^	0.214^a^
Hydroponic experiment	62d	MS	6.75^c^ ± 0.12	8.26^b^ ± 0.39	0.85^c^ ± 0.04	1.18^b^ ± 0.09	0.126^c^	0.144^b^
		IS	8.57^b^ ± 0.25	10.33^a^ ± 0.46	1.24^b^ ± 0.15	2.02^a^ ± 0.11	0.145^b^	0.195^a^
		MM	7.49^c^ ± 0.73	9.43^b^ ± 0.16	1.93^d^ ± 0.12	2.13^c^ ± 0.13	0.259^a^	0.226^b^
		IM	9.68^b^ ± 0.42	12.73^a^ ± 0.18	2.49^b^ ± 0.15	2.78^a^ ± 0.09	0.257^a^	0.219^b^

Numbers were Mean ± SD or Mean. Different lowercase letters denoted significant difference between monocropped and intercropped treatments under different *P* levels (*P* ≤ 0.05).

MS, monocropped soybean; IS, intercropped soybean; MM, monocropped maize; IM, intercropped maize.

### Soybean nodulation

Intercropping significantly stimulated soybean nodulation compared to monocropping in both soil and hydroponic experiments (Table 2). On the 75th d in the soil experiment, nodule number and nodule dry weight of intercropped soybean under both deficient and sufficient P levels increased by 73.91%, 82.95% and 99.56%, 62.70% respectively, compared to monocropped soybean (Table 2). Also in the hydroponic experiment (62th d), intercropping significantly increased the number and dry weight of nodules on soybean roots by 64.00%, 56.41% and 30.10%, 50.64% at two levels of P supply, respectively (Table 2). However, soybean nodulation was significantly inhibited by low P supply in both soil and hydroponic experiments, which might be explained by the observed significant decline in the number and dry weight of soybean nodules.

**Table 2:** Nodulation of soybean as affected by intercropping and P levels.

Experiments	Times (days)	Cropping patterns	Nodule number/NO plant^−1^		Nodule dry weight/g plant^−1^	
			Deficient-P	Sufficient-P	Deficient-P	Sufficient-P
Soil experiment	75	MS	23^d^ ± 0.90	32^c^ ± 0.74	0.132^d^ ± 0.00	0.191^c^ ± 0.01
		IS	40^b^ ± 1.18	59^a^ ± 1.29	0.263^b^ ± 0.01	0.312^a^ ± 0.01
Hydroponic experiment	62	MS	25^d^ ± 1.53	39^c^ ± 1.75	0.196^c^ ± 0.01	0.233^b^ ± 0.01
		IS	41^b^ ± 1.55	61^a^ ± 1.27	0.255^b^ ± 0.02	0.351^a^ ± 0.02

Numbers were Mean ± SD. Different lowercase letters indicated significant difference between monocropped and intercropped treatments under different P levels (*P* ≤ 0.05).

MS, monocropped soybean; IS, intercropped soybean.

**Figure 1:**
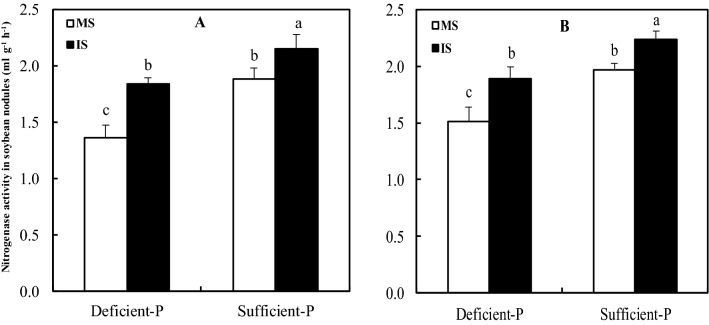
Nitrogenase activity of soybean nodule as affected by intercropping and P levels. MS, monocropped soybean; IS, intercropped soybean. (**A**) Soil experiment, (**B**) hydroponic experiment. Different lowercase letters indicated significant difference between monocropped and intercropped treatments under different P levels (*P* ≤ 0.05).

Nitrogenase activity of nodules is one of indices assessing N_2_ fixation efficiency of leguminous plants. Nitrogenase activity in soybean nodules was frequently enhanced when intercropped with maize than grown alone at two levels of P supply (Fig. 1). In both soil and hydroponic experiments, the nitrogenase activity in intercropping-induced nodules significantly increased by 35.29%, 14.36% and 25.17%, 13.71% under both deficient and sufficient P levels than monocropping, respectively. Nevertheless, the nodule nitrogenase activities of either inter- or mono-cropped soybean grown under deficient P condition were decrease in both soil and hydroponic experiments.

### P levels in soybean leaves, nodules and roots

**Figure 2:**
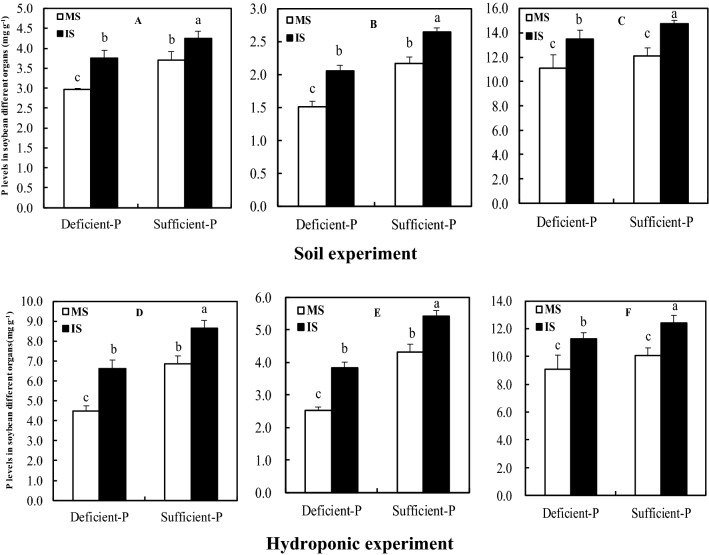
P concentration in soybean different organs as affected by intercropping and P levels. MS, monocropped soybean; IS, intercropped soybean. (**A**,**D**) Leaves, (**B**,**E**) roots, (**C**,**F**) nodules. Different lowercase letters indicated significant difference between monocropped and intercropped treatments under different P levels (*P* ≤ 0.05).

P levels in nodules of intercropped soybean under both deficient and sufficient P levels increased by 15.08% and 22.07% in the soil experiment (75th d) respectively, compared to monocropped soybean (Fig. 2). Similarly, the P levels in intercropping-induced nodules supplied with two P levels enhanced by 19.47% and 23.19% on the 62th d in the hydroponic experiment. Also in leaves and roots, P levels of intercropped soybean in both soil and hydroponic experiments were higher than that in monocropped soybean.

Low P supply resulted in a significant decrease in P levels in leaves and roots of intercropped- or monocropped-soybean in both soil and hydroponic experiments (Fig. 2); the reduction in the soil experiment (11.53% and 20.22% for leaves, 22.64% and 30.41% for roots) and the reduction in the hydroponic experiment (23.26% and 34.5% for leaves, 29.28% and 41.80% for roots); whereas there were no significant decline in nodular P levels. P level in nodules of soybean whether deficient- or sufficient-P conditions remarkably greater than those in leaves and roots.

### Acid phosphatase (APase) and phytase activities in soybean nodules, roots, and leaves

**Figure 3:**
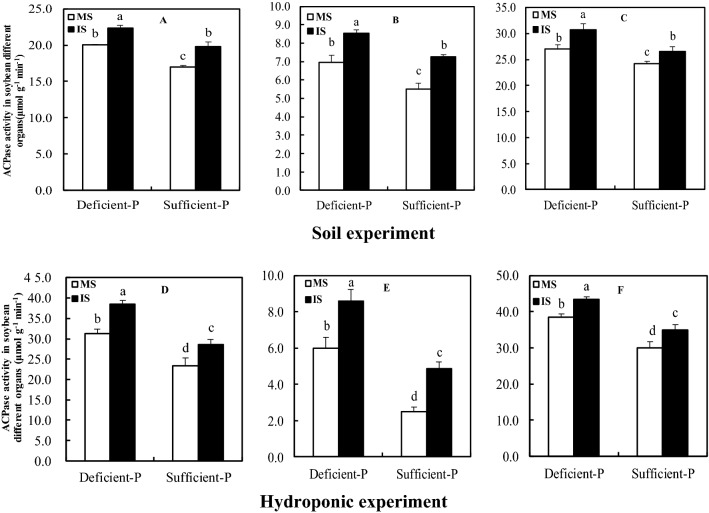
APase activity in soybean different organs as affected by intercropping and P levels. MS, monocropped soybean; IS, intercropped soybean. (**A**,**D**) Leaves, (**B**,**E**) roots, (**C**,**F**) nodules. Different lowercase letters indicated significant difference between monocropped and intercropped treatments under different P rates (*P* ≤ 0.05).

**Figure 4:**
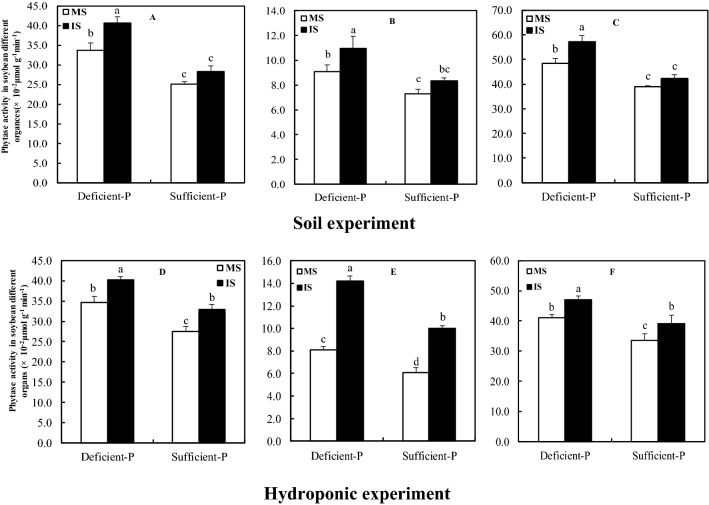
Phytase activity in soybean different organs as affected by intercropping and P levels. MS, monocropped soybean; IS, intercropped soybean. (**A**,**D**) Leaves, (**B**,**E**) roots, (**C**,**F**) nodules. Different lowercase letters mean the significant difference between monocropped and intercropped treatments under different P levels (*P* ≤ 0.05).

Induction of key enzymes involved in improvement of internal P utilization efficiency displayed a profound variation in response to cropping patterns and P supply (Figs. 3 and 4). On the 75th d in the soil experiment, APase activities in nodules, leaves and roots of intercropped soybean under both deficient and sufficient P levels significantly enhanced by 13.72% and 9.76%, 11.68% and 16.68%, 22.74% and 32.24% than monocropping, respectively, as well as the phytase activities (18.28% and 8.40%, 20.21% and 12.29%, 20.88% and 13.84%). Also in the hydroponic experiment (62th d), intercropping caused a significant enhancement in the activities of APase and phytase in nodules, leaves and roots relative to monocropping at two levels of P supply.

Reduced P supply resulted in a significant increase in the activities of APase and phytase in nodules of either inter-or mono-cropped soybean, as well as their leaves and roots. And the activity of APase and phytase in nodules much higher than those in leaves and roots. Particularly, the activities of APase and phytase in nodules of intercropped soybean supplied with deficient P level reached the maximum, as well as leaves and roots.

### Expression of phytase gene in soybean nodules and roots

**Figure 5:**
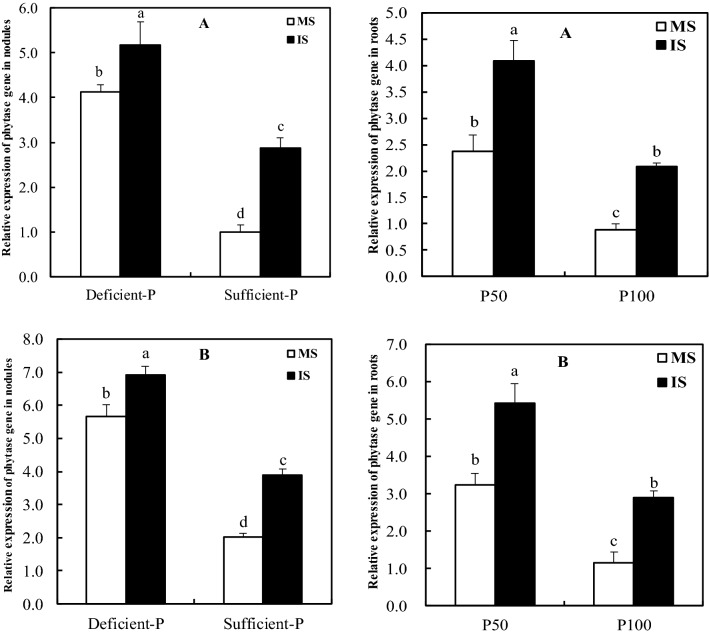
Relative expression of phytase gene in soybean nodules and roots as affected by intercropping and P levels. MS, monocropped soybean; IS, intercropped soybean. (**A **) Soil experiment, (**B **) hydroponic experiment. Different lowercase letters indicated significant difference between monocropped and intercropped treatments under two P levels (*P* ≤ 0.05).

Expression level of phytase gene in nodules and roots of soybean was significantly induced by intercropping under both deficient-and sufficient-P levels (Fig. 5), as indicated by 25.7%, 188.0% and 72.6%, 137.5% increases in soil experiment compared with soybean grown alone, respectively. Also in hydroponic experiment, the expression level of phytase gene in nodules and roots increased by 22.1%, 164.6% and 67.6%, 151.3% relative to monocropped soybean, respectively. Furthermore, a reduction in P supply significantly resulted in higher expression levels of phytase gene in nodules and roots of either inter-or mono-cropped soybean in both experiments, and the maximum expression levels was observed in intercropped soybean supplied with deficient P supply. In addition, the expression level of phytase gene was higher in nodules than in roots.

## Discussions

Legume/cereal intercropping is widely practiced as a sustainable food-production pathway in tropical and temperate regions^40^ due to it could reduce the input of fertilizers by symbiotic N_2_ fixation of associated legume^41^. Much present work have demonstrated that a low soil N availability and abundant root exudates were the primary mechanisms driving increased nodulation in legume/cereal intercropping systems^23,42^. In our experiments, the nodulation and N_2_ fixation in soybean were enhanced when intercropped with maize than grown alone, even under low P fertilization, which might be explained by the significant increase in the number, dry weight and nitrogenase activity of nodules (Table 2). Our results were in line with results of Li et al.^23^ and Liu et al.^21^ on nodulation and N_2_ fixation, who reported that the enhancement of nodulation was likely caused by the enrichment flavonoids in root exudates in faba bean/maize and faba bean/wheat intercropping systems, but the detailed mechanisms underlying how intercropping regulate nodulation and N_2_ fixation of legumes under P deficient level, is largely unknown. Then, improved nodulation of soybean in intercropping under P deficient condition facilitated the growth of soybean and neighbouring maize (Table1).

The high N_2_ fixation efficiency of legumes would largely depend on efficient P allocation and the elevated P concentrations in nodules, particularly under P stressful environments^43,44^. Up to 20% of total plant P was preferentially partitioned to nodules to support nodule efficient N_2_ fixation, even much higher P under phosphorus starvation conditions^45^. P concentrations in legume nodules were remarkably higher than that in any other organs to improve N_2_ fixation efficiency, even over three-folds undergoing P-limited conditions^15^. Our experiments also found that P levels in nodules of soybean supplied with sufficient- or deficient-P levels apparently greater than that in leaves or roots (Fig. 2), even up to 10-folds. These data implied that nodule was a preferential strong P sink with an ability of maintaining the nodular P homeostasis during P scarcity.

An apparent reduction of P levels in roots and leaves of soybean under P deficiency, but not in nodules (Fig. 2) agreed with previous observations of Jebara et al., who reported that P deficiency less affected P concentration in common bean nodules, but significantly decreased P concentration in shoots and roots^45^. Interestingly, however, intercropped with maize could significantly increase P levels in nodules, roots and leaves in soybean compared with monocropped soybean whether deficient-and sufficient-P levels (Fig. 2), it's presumably because root-root interaction of soybean and maize showed an interspecific facilitation that more efficiently improve internal P contents than monocropped soybean, as evidenced by our previous results^46^. More interestingly, P level in nodules of intercropped soybean supplied with deficient P was significantly higher than that in nodules of monocropped soybean with P sufficient level. These observed results in present experiments together suggested that intercropped with maize could contribute to soybean adaptation during P scarcity by interspecific facilitation.

Phosphatase and particularly phytase are involved in the enhancement of P uptake and its internal use and recycling within the plant tissues^47,48^. The same results were made in some studies on common bean nodules^30,49,50^, which suggested that these plant phytase and APase were most likely involved in P homoeostasis in the nodules under P starvation. In present study, the enhanced activities of phytase and APase in nodules of either monocropped or intercropped soybean were observed under low P supply, revealing phytase and APase might be involved in the improvement of P level in nodules, and agreed with previous observations on common bean and faba bean^51,52^. Remarkably, the elevated expression level of phytase gene in the nodules and roots under P deficiency and its expression level was higher in nodules than in roots (Fig. 5), coincided with the increased activities of nodular phytase and APase (Figs. 3 and 4), indicating the involvement of this particular gene in nodular P homoeostasis by enhancing the activities of phytase and APase, and agreed with results of Araújo et al.^30^ and Lazali et al.^32^ on common bean.

Indeed, greater root exudation of acid phosphatase by legumes is one of drivers increasing P acquisition in legume/cereal intercropping systems^17,53^. In our studies, intercropped with maize could significantly increase the activities of phytase and APase, and expression level of phytase gene in soybean nodules as compared to monocropped soybean (Figs. 3, 4 and 5), revealing that soybean/maize intercropping also could induce the synthesis of phytase and APase in nodules. Furthermore, the maximum activities of phytase and APase as well as the highest expression of phytase gene in nodules of intercropped soybean supplied with low P were observed relative to other treatments (Figs. 3, 4 and 5). These findings in present experiments comprehensively elucidated that the enhancement of P level in nodules was most likely caused by improvement of phytase and APase activities when soybean intercropped with maize under P deficient conditions.

## Conclusions

Our results suggest that the enhanced nodulation in soybean/maize intercropping system under P deficiency induced by elevated P level in nodules, as compared to monocropped soybean. The increased activities of phytase and acid phosphatases in intercropping-induced nodules under low supply of P fertilizer were considered as one of important forces driving enhancement in nodular P level. The expression of phytase gene was positively associated with the activities of phytase and APase in nodules. We concluded that the expression and activity of phytase in nodules under low P conditions played a pivotal role in symbiotic N_2_ fixation, but the quantitative expression of the genes involving in the nodule function should be further investigated. Our results would advance our understanding on the underlying mechanisms of interspecific facilitation on nodulation and N_2_ fixation in legume/cereal intercropping systems, particularly under P stressful environments.
